# Effects of pH and Ionic Salts on the Emulsifying and Rheological Properties of Acorn Protein Isolate

**DOI:** 10.3390/molecules27113646

**Published:** 2022-06-06

**Authors:** Nasir Mehmood Khan, Muhammad Saeed, Farman Ali Khan, Shujaat Ahmad, Muhammad Asif Nawaz, Zia Ullah Khan, Muhammad Shafique, Mazen Almehmadi, Osama Abdulaziz, Abid Ullah

**Affiliations:** 1 Department of Agriculture, Shaheed Benazir Bhutto University, Sheringal, Dir Upper 18000, KP, Pakistan; nasir@sbbu.edu.pk; 2 Department of Chemistry, Shaheed Benazir Bhutto University, Sheringal, Dir Upper 18000, KP, Pakistan; saeedsawati911@gmail.com (M.S.); or farmanali@sbbu.edu.pk (F.A.K.); 3 Department of Pharmacy, Shaheed Benazir Bhutto University, Sheringal, Dir Upper 18000, KP, Pakistan; shujaat@sbbu.edu.pk (S.A.); abid@sbbu.edu.pk (A.U.); 4 Department of Biotechnology, Shaheed Benazir Bhutto University, Sheringal, Dir Upper 18000, KP, Pakistan; asif_biotech33@yahoo.com; 5 Department of Food Science and Technology, Abdul Wali Khan University, Mardan 23200, KP, Pakistan; ziaullahkhan@awkum.edu.pk; 6 Department of Pharmaceutical Sciences, College of Pharmacy-Boys, Al-Dawadmi Campus, Shaqra University, Shaqra 15571, Riyadh, Saudi Arabia; 7 Clinical Laboratory Sciences Department, College of Applied Medical Sciences, Taif University, Taif 26513, Makkah, Saudi Arabia; dr.mazen.ma@gmail.com (M.A.); o.osama@tu.edu.sa (O.A.)

**Keywords:** API (acorn protein isolate), rheology, emulsifying properties, solubility, ionic salts

## Abstract

This study was designed to evaluate the emulsifying and rheological properties of acorn protein isolate (API) in different pH mediums (pH 3, 7 and 9) and in the presence of ionic salts (1 M NaCl and 1 M CaCl_2_). API shows higher solubility in distilled water at pH 7, while at the same pH, a decrease in solubility was observed for API in the presence of CaCl_2_ (61.30%). A lower emulsifying activity index (EAI), lower stability index (ESI), larger droplet sizes and slight flocculation were observed for API in the presence of salts at different pHs. Importantly, CaCl_2_ treated samples showed relevantly higher EAI (252.67 m^2^/g) and ESI (152.67 min) values at all pH as compared to NaCl (221.76 m^2^/g), (111.82 min), respectively. A significant increase in interfacial protein concentration (4.61 mg/m^2^) was observed for emulsion at pH 9 with CaCl_2_, while the major fractions of API were observed in an interfacial layer after SDS-PAGE analysis. All of the emulsion shows shear thinning behavior (τ_c_ > 0 and *n* < 1), while the highest viscosity was observed for emulsion prepared with CaCl_2_ at pH 3 (11.03 ± 1.62). In conclusion, API, in the presence of ionic salts at acidic, neutral and basic pH, can produce natural emulsions, which could be substitutes for synthetic surfactants for such formulations.

## 1. Introduction

Plant proteins are underutilized in the food industry because of their poor structural and functional properties in various products in unmodified forms. Recently, some advanced methods were adopted to modify the functional properties of such proteins, including the alternation in molecular structure or changing the environment of protein functionalities [1]. Plant proteins are reported to stabilize emulsions which have a critical role in a wide range of food applications [2]. Emulsion is the mixture of two immiscible liquids where proteins act as surfactants by reducing the interfacial tension [3]; however, all of the relevant properties of an emulsion depend upon the protein molecular structure, solubility and medium of conditions, i.e., temperature, pH and ionic salts concentration [4].

Oak trees (Quercus) are evergreen plants and are found in various parts of the world [5]. The fruit of the oak tree is known as acorn and has extensively been studied for its nutritional importance [6]. In Pakistan, the acorn is considered a wild fruit that is wasted directly, which results in agricultural byproducts. Such byproducts are usually composed of proteins, fibers and other important molecules. Acorn protein could be a cheap and sustainable alternative to animal proteins (eggs and meat) due to its availability as well as its role as a value-added byproduct from acorn waste. Moreover, acorn protein possesses potential emulsifying properties [7], which need to be explored further since no previous work has reported on this protein. Acorn fruit has been labelled as new healthy food because it is one of the richest sources of vitamins, minerals, starch, protein and fibers as compared to other cereals [8]. Acorn proteins consist of essential amino acids with better nutritional values as compared to other cereal proteins, probably due to higher lysine composition [9]. The lysine has a linear structure which makes it more suitable for emulsifying properties in product development [7]. In many food formulations, it is important to control the composition of various ingredients, i.e., in infant formula and beverages industries [10]. Different pH and ionic salt concentrations have great contributions to such food formulation; therefore, the current study was designed in order to sketch the various functional characteristics of acorn protein isolate in the presence of various ranges of pH and ionic salt concentrations. Recently, emulsifying properties of acorn protein isolates have been reported with effects of different ratios of oil and protein concentration [7]; however, the study on acorn protein emulsifying properties is limited and needs to be evaluated in-depth to understand the utilization of this protein in food industries for the development of various food products.

## 2. Materials and Methods

### 2.1. Acorn Flour Preparation

Acorn fruits were collected from nearby mountains on the campus. Mature and undamaged fruits were separated and blended without further addition. The blended fruit was mixed with n-hexane to remove oil contents, followed by stirring for 3 h at 25 °C and vacuum filtration. The obtained flour over the filter paper was dried in the oven at 40 °C for 48 h and was ground to a fine powder for further analysis.

### 2.2. Protein Extraction from Flour

The dried flour was used for API extraction as previously described by Aziz et al. [7] with some modifications. Briefly, 200 g of flour was mixed with 2 L deionized water, and the pH was adjusted to 11.0 using 5 N NaOH. After 3 h magnetic stirring, the mixture was centrifuged at 20,000 rpm for 20 min at room temperature. The supernatant was collected, and its pH was adjusted to 4.5 using 5 N HCl followed by centrifugation. The precipitate was washed with deionized water and collected from the tubes. The precipitate was dissolved in distilled water, adjusted to pH 7.0 using 5 N NaOH and then lyophilized. The following composition was identified as per standard protocols [11], 91.02% protein, 0.31% fat, 0.57% crude fiber, 4.0% ash and 4.1% moisture.

### 2.3. API Solubility

API solubility was measured according to the method reported by Casella and Whitaker [12]. In total, 1 M NaCl or 1 M CaCl_2_ was added to a 1% API (*w*/*v*) solution and stirred for 1 h. Afterwards, the pH was adjusted to 3.0, 7.0 and 9.0 with 0.5 M HCl or NaOH, and then samples were centrifuged at 5000 rpm for 20 min. The pellet was discarded, and the API concentration in the supernatant was measured by the colorimetric method [13,14]. Bovine serum albumin was used as the standard. Solubility was calculated as Equation (1):

(1) $$\frac{Csup}{CAPI}\times 100$$

where *Csup* is the protein content of supernatant, and *CAPI* is the protein content in the API solution.

### 2.4. Emulsifying Activity Index (EAI) and Emulsifying Stability Index (ESI)

The standard method proposed by Pearce and Kinsella [15] was used to determine the EAI and ESI. A 1 % (*w*/*v*) API solution was developed in distilled water, and different pH ranges were adjusted, i.e., pH 3.0, 7.0 and 9.0 with and without the addition of 1 M NaCl or 1 M CaCl_2_ to each of the solutions separately. The emulsion was prepared by homogenizing the API solution and corn oil (3:1) using a D-160 DLABT homogenizer with a 12 mm head (China) for 1 min at 30,000 rpm. In total, 20 µL of each freshly prepared emulsion was diluted with 5 mL of 0.1% (*w*/*v*) sodium dodecyl sulfate (SDS) solution and was shaken rapidly. The role of SDS in this method is to maintain and control the coalesce and droplet size distribution. It is used to obtain the individual particle size (diameter). The absorbance was measured at 500 nm using a spectrophotometer (model, manufacturer, country). For the determination of ESI, the homogenized sample was kept for 10 min at room temperature, and then the absorbance was recorded as earlier. The following Equations (2) and (3) were used for the calculation of EAI and ESI.

(2) $$\mathrm{EAI}\;\left(m^{2}/g\right)=\frac{2\times 2.303\times A_{0}\times \mathrm{dilution}\;\mathrm{factor}}{c\times 1\times \left(1-\varphi \right)\times 10,000}$$

(3) $$\mathrm{ESI}\;\left(\min\right)=\frac{A_{0}}{A_{0}-A_{10}}\times t$$

c = correspond to actual protein amount (g/mL); l = present cuvette path length (0.01 m); φ = present ration between protein and oil (0.25); dilution factor (250); A_0_ = initial absorbance at zero time, A_10_ = emulsion absorbance after 10 min of storage.

### 2.5. Emulsion Microphotograph

A microphotograph of all the emulsions was captured using a SMT 3 microscope (China). A very small amount of emulsion (20 µL) was placed on a glass slide without adding any dye or chemicals, and photos were taken to study flocculation and droplet distributions. The same emulsion was used for the microphotograph after 2 h to study coalescence.

### 2.6. Droplets Mean Diameter

The volume-weighted mean diameter (D_4,3_) and volume surface mean diameter (D_3,2_) of the emulsions were determined according to Aziz et al. [7]. The freshly prepared emulsion was diluted with 1% SDS solution (10 mL), and mean diameters were calculated using a Mastersizer Particle Size Analyzer (Mastersizer 2000, Malvern Instruments, Worcestershire, UK). The following Equation (4) was used to calculate the emulsion specific surface area according to Walstra [16]. SDS maintains and controls the coalesce and droplet distribution. It is used to obtain the individual particle size (diameter).

Sv = 6φ/D_3,2_ (m^2^/mL emulsion) (4)

φ = present ratio between protein and oil.

### 2.7. Concentration of API at the Oil–Water Interface

The protein concentration at the oil–water interface was determined according to the method of Patton and Huston [17]. Briefly, the freshly prepared emulsion was mixed with sucrose solution (2:2) and carefully released in a centrifuged tube containing 7 mL of sucrose solution. The mixture was centrifuged at 5000 rpm for 30 min. After centrifugation, the tubes were kept in a refrigerator for 24 h at −40 °C. The top cream layer was cut through a sharp blade and mixed with SDS solution (1% *w*/*v*). The concentration of API at the oil–water interface was determined in mg/mL according to Markwell et al. [14]. The following Equation (5) was used to calculate Г (mg/m^2^).

Г = Cad/Sv(5)

Sv = Emulsion droplet specific interfacial area (m^2^/mL emulsion).

### 2.8. Composition of the Oil–Water Interface at SDS-PAGE

The composition of the interfacial layer was evaluated by SDS-PAGE according to the protocols described by Laemmli [18]. The electrophoretic pattern of protein adsorbed to the oil droplet was compared to the standard marker. The gel was conducted with 5% stacking gel and 15% running gel using a discontinuous buffer system. A ladder with molecular weight markers of 14.4–97.4 kDa was used (Sigma Aldrich, Darmstadt, Germany).

### 2.9. Emulsion Rheological Measurement

Immediately after homogenization, 1 mL of the emulsion was loaded to the Physica MCR 301rheometer (Anton Paar, Graz, Austria) equipped with a cone/plate system. The samples were allowed to sit for temperature equilibrium and relaxation (25 °C). Afterwards, the shear rate was adjusted to obtain a flow curve from 0.01 to 1000 s^−1^ for 2 min. The flow curves of emulsions were evaluated by Herschel–Bulkey’s model (6).

τ = τ_c_ + k·γ^n^ (6)

where τ_c_ = yield value (Pa), k = viscosity coefficient (Pa s^n^), ˙γ = shear rate (s^−1^) and *n* = flow index. A measure of the viscosity (η, in Pa s) was given at a shear rate (˙γ) of 125 s^−1^ for all the samples.

### 2.10. Statistical Analysis

Each experiment was repeated three times. The data were analyzed using a one-way ANOVA. The means were compared by the Duncan multiple range test at *p* < 0.05 using SAS software.

## 3. Results and Discussion

### 3.1. API Solubility in the Presence of NaCl and CaCl_2_

**Figure 1:**
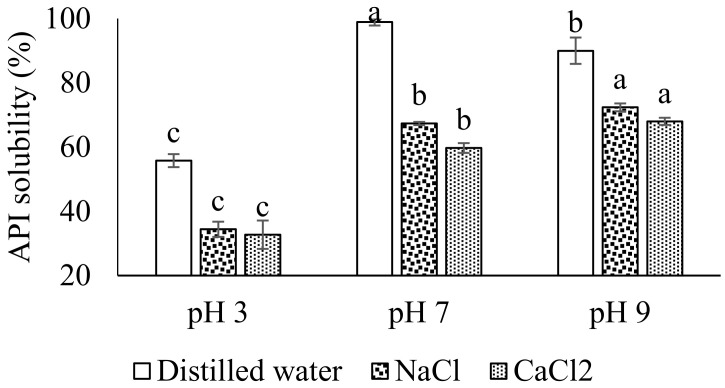
Solubility profile of API at different pH mediums in distilled water and in the presence of NaCl and CaCl_2_. Different letters (a–c) represent significant differences among data. The significant difference was calculated individually among each treatment.

The API solubility in the presence of NaCl and CaCl_2_ is shown in Figure 1. The solubility profile of any protein is important for its functional characteristics, particularly in emulsion formulations [7]. As protein has a wide range of industrial applications, protein should be evaluated for its solubility in the presence of ash contents, minerals, vitamins and ionic salts, etc., for specific functional purposes [19]. In distilled water, the solubility profile remained significantly higher at pH 7, while low solubility of API was observed at pH 3. Further, the API showed significantly higher (*p* < 0.05) solubility at pH 9 both in the presence of NaCl and CaCl_2_, followed by pH 7 and pH 3. The higher solubility of API in the presence of ionic salts at pH 9 and 7 is probably because the protein-positive and negative net charged molecules interact more with water, while at pH 3 there might be lower electrostatic forces which render most of the protein water interactions [20]. It is obvious that proteins show higher solubility above their isoelectric point in distilled water, where intermolecular repulsion enhances solubility by negative and positive charges. However, the addition of NaCl and CaCl_2_ to the API solution at different pHs showed a significant decrease in solubility. At pH 3, solubility decreased up to 17.62% and 18.81% with the addition of NaCl and CaCl_2_, respectively. Similarly, at pH 7, the decrease was observed up to 58.44% and 61.30%, while at pH 9, a decrease of 46.98% was noted with the addition of salts. The decrease in solubility of API is probably because of the NaCl and CaCl_2_ salting-out effect [21]. It was interesting to observe that the API solubility was influenced more prominently at pH 7 as compared to pH 3 and pH 9. The higher decrease at pH 7 is probably because protein has different charged groups, which defines its solubility as per available pH and type of salts.

Similar results for protein solubility have been reported for sweet potato and chicken meat proteins [20,21].

### 3.2. EAI and ESI

**Figure 2:**
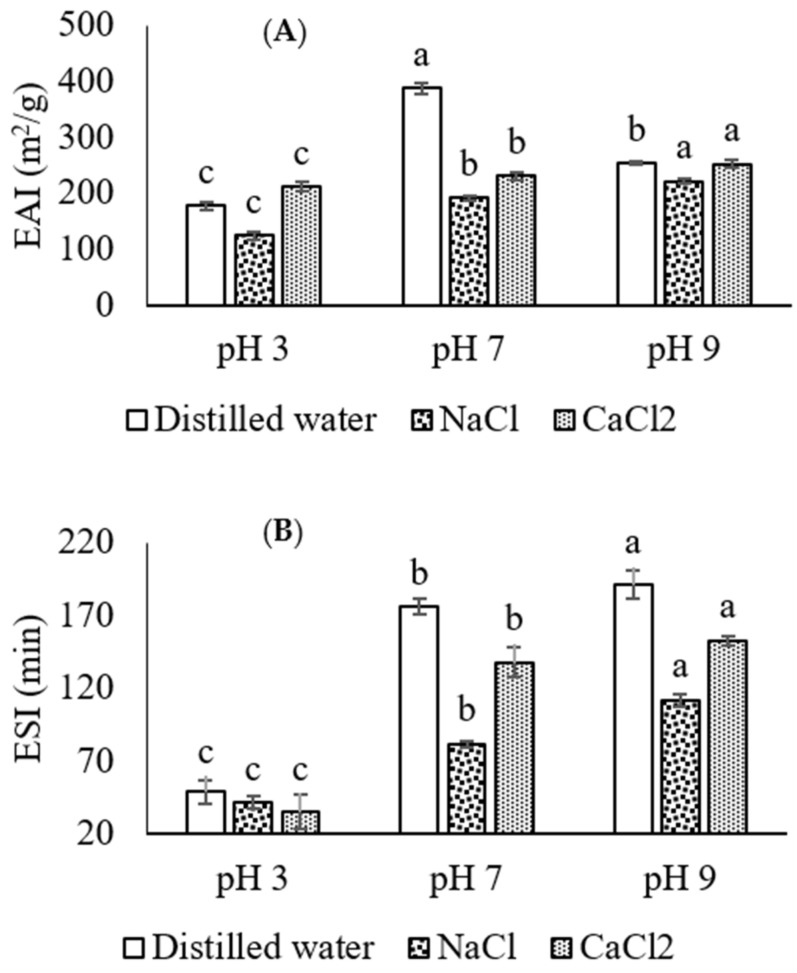
Emulsifying activity index (**A**) and emulsifying stability index (**B**) of API at different pH mediums in distilled water and in the presence of NaCl and CaCl2. Different letters (a–c) represent significant differences among data. The significant difference was calculated individually among each treatment.

The EAI and ESI of API emulsions are shown in Figure 2A,B. The emulsifying properties of API in distilled water, as well as with the addition of salts at different pH, shows scattered values for their respective EAI and ESI. The study on fenugreek protein isolate suggests that higher solubility of protein is a key factor that enhances emulsifying properties [22]. In the current study, such a trend for EAI was only observed for pH 7 in distilled water, showing 387.9 (m^2^/g) values with higher solubility (98.93%) values. Our results for EAI at pH 3 and 9 are not in agreement with the solubility profile of API both in the distilled water and salt added samples (Figure 1 and Figure 2A). The ESI of API in distilled water and in the presence of salts is shown in Figure 2B. The significantly higher (*p* < 0.05) stability of emulsion was observed for pH 9 in distilled water having 190.9 (min), followed by CaCl_2_ (152.67 (min)) and NaCl (111.82 (min)), respectively. All of the emulsion stabilities remained dependable upon the nature of pH and or addition of salts, particularly the addition of NaCl or CaCl_2_ at pH 3, 7 and 9 reduced the stability of the emulsion. Furthermore, significantly (*p* < 0.05) lower ESI values were observed at pH 3 where the reduction in ESI of API was 59% in distilled water, 46% in NaCl and 63% in CaCl_2_, respectively. The modification in emulsifying properties of API is probably because of changes in pH, and the addition of salts could denature the protein structure. These results suggest that the charge and ionic salts reduce the emulsifying properties of API due to the alternation or denaturation of the API structure. Additionally, ionic salts are vital ingredients in many emulsions, i.e., butter, milk, cream, soup and mayonnaise. API could be one of the substitutes for synthetic surfactants in such formulations in the presence of NaCl and CaCl_2_ at different pH conditions.

### 3.3. Droplet Size and Emulsion Microscopy

**Figure 3:**
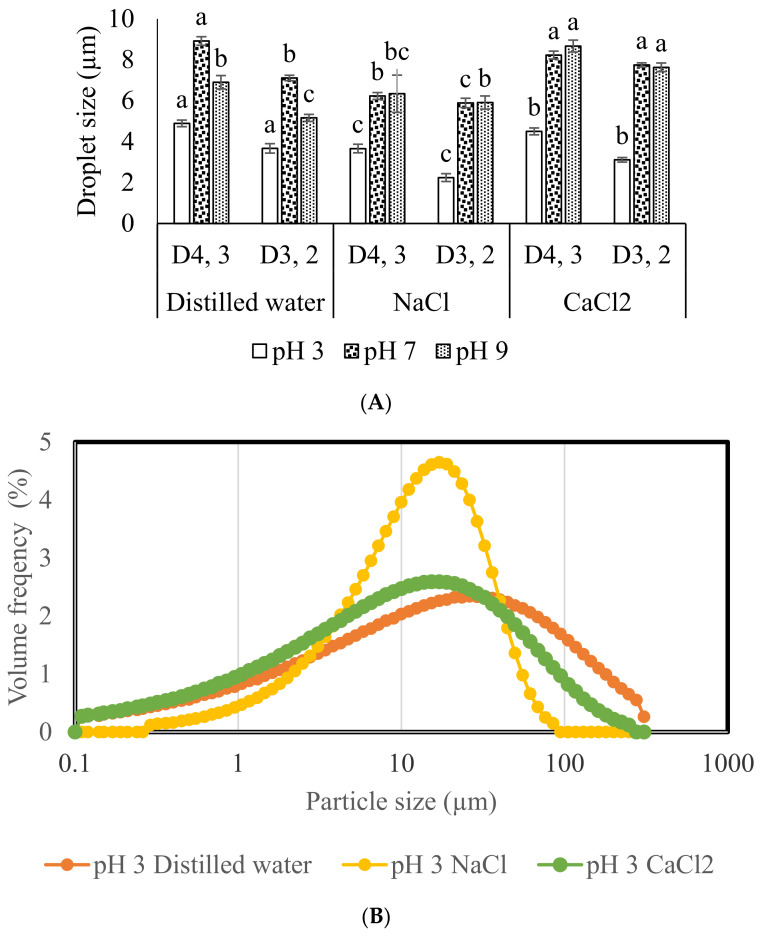

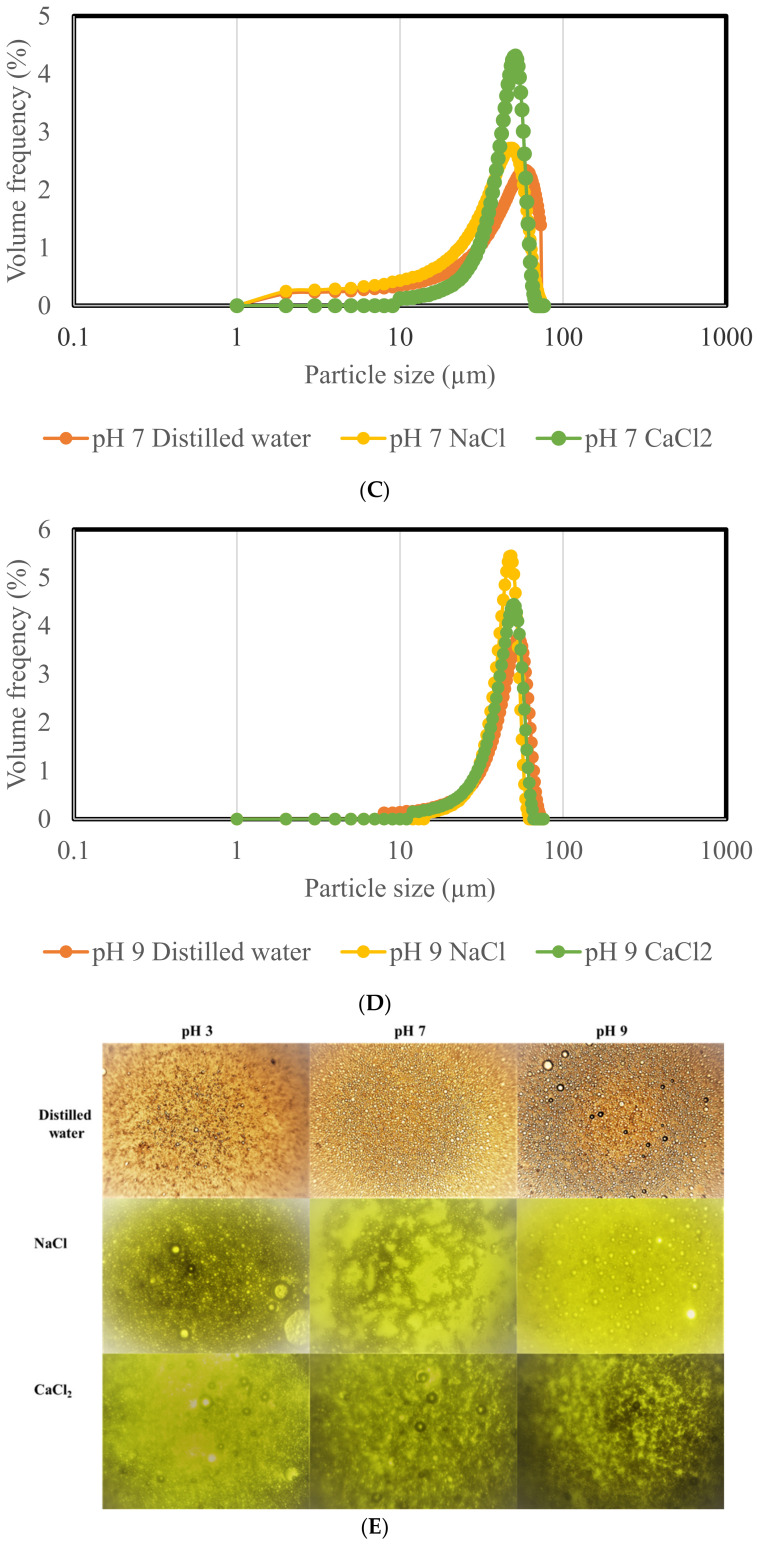

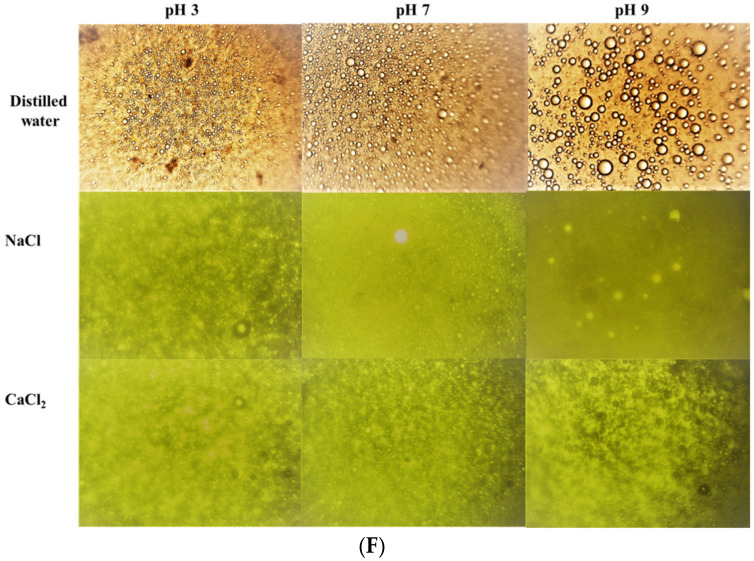
Droplet sizes of API emulsion (**A**) at different pH mediums in distilled water and in the presence of NaCl and CaCl_2_, emulsion particle size distribution (**B**–**D**), emulsion photograph at zero time (**E**) and after 2 h storage (**F**). Different letters (a–c) represent significant differences among data. The significant difference was calculated individually among each treatment.

The droplet sizes of API emulsion prepared at pH 3, 7 and 9 in distilled water and with ionic salts are shown in Figure 3A. The droplet size study showed variation in their values in distilled water as well as in the presence of ionic salts. Significantly larger droplet sizes (D_4,3_ and D_3,2_) were observed for API emulsion in distilled water at pH 3 followed by pH 9 and 7. A similar case was noted with the addition of salts at different pH, respectively. The larger droplet size of emulsions in the presence of salts at different pH is probably because of the slight flocculation, which may be attributed to the weak repulsive forces between droplets [23]. Some of the studies reported different emulsion droplet sizes as a function of salt concentration. Milk protein stabilized emulsion has been reported to show constant droplet sizes with the addition of NaCl [24]; in addition, other studies reported an increase in emulsion droplet size for protein extracted from microalgae [25]. The significantly larger droplet sizes in this study in the presence of salts indicate the destabilization of oil droplets due to a reduction in charge and solubility. This hypothesis correlates with the solubility profile of API, as noted in Figure 1. However, many of the studies reported that the addition of salt to emulsion shielded the electrostatic screening effects and therefore decreased the electrostatic repulsion between droplets which favor higher D_4,3_ and D_3,2_ [26]. This hypothesis was also confirmed by the volume frequency vs. particle size distribution of larger droplet sizes (Figure 3B–D). The droplet size distribution for pH 3 with and without ionic salts is shown in Figure 3B, where the highest volume frequency of particle size distribution was observed for NaCl (4.62%), followed by CaCl_2_ (2.58%) and distilled water (2.32%), respectively. A similar increase in droplet size curve was observed at pH 7 and pH 9 (Figure 3C,D) with the addition of salts, where a higher volume frequency distribution was observed for pH 9 with NaCl (5.32%). These results indicate that the electrostatic screening effects of salts results in larger droplet sizes. Furthermore, it is obvious that smaller droplet sized emulsion presents higher EAI values; however, the increase in droplet size has reverse effects. The lower EAI values for API emulsion in this study (Figure 2A) confirmed that the addition of salts at different pH values increases the volume frequency of larger droplets and consequently decreases the EAI capabilities of emulsions.

Emulsion photographs are shown in Figure 3E,F. The photos were taken from freshly prepared emulsions (Figure 3E) and after 2 h storage of the emulsion (Figure 3F). Emulsion microscopy mostly defines the distribution, flocculation and coalescence factor among oil droplets. The freshly prepared emulsions of API in distilled water at pH 3, 7 and 9 show the uniform distribution of the oil droplets, where initially, no flocculation and or any coalescence was observed. However, larger size oil droplets were observed after 2 h storage of the same emulsion (Figure 3F), which was possible because of the slight coalescence, where oil droplets colloid with each other and convert into larger sizes. The emulsions of API in the presence of NaCl show flocculation initially, particularly at pH 7, while pH 3 and 9 show uniform distribution (Figure 3E). The storage of the same emulsion for 2 h shows a significant reduction in the number of oil droplets where dense emulsions were observed for NaCl at pH 3, 7 and 9, which was possible because of the immersion of oil droplets in the aqueous phase or the slow demolishing of the interface (Figure 3F). The API emulsions in the presence of CaCl_2_ describe the same phenomena as noted with NaCl at pH 3, 7 and 9. The impact of salt on the emulsifying behavior of protein is quite complex, depending upon the accurate nature of minerals ions involved [27]. Furthermore, the pH far from the isoelectric point with the addition of salts causes a reduction in electrostatic repulsion among protein molecules which leads to the aggregation of protein in the emulsion. In our previous study, API showed an homogenous structure of oil droplets with different oil and protein concentrations [7]; however, in this study, we concluded that variation in pH and the addition of salt affect the API emulsion characteristics probably because of the alternation in protein structure in such an environment.

### 3.4. Interfacial Protein Concentration (Г)

**Figure 4:**
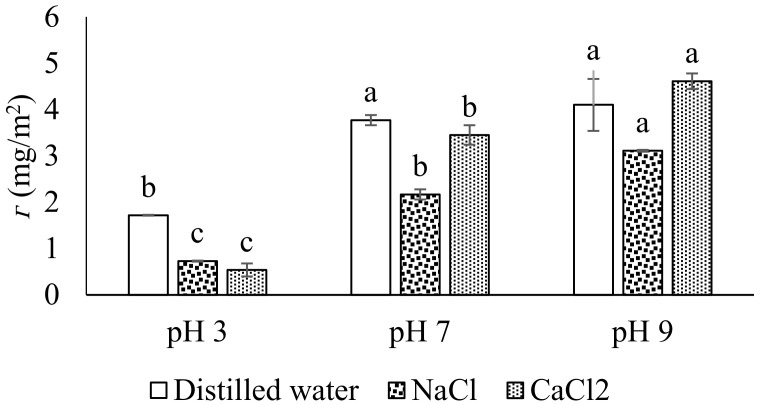
Interfacial protein concentrations of API at different pH mediums in distilled water and in the presence of NaCl and CaCl_2_. Different letters (a–c) represent significant differences among data.

The emulsion interfacial protein concentrations are shown in Figure 4. An interfacial layer of oil-in-water emulsion is a key parameter that determines the stability of emulsion, i.e., the higher the concentration, the higher the stability would be. However, other factors such as the droplet size, medium of pH and presence of ionic salts also influence the stability either positively or negatively. The results reveal a significantly lower value of Г (1.72 for distilled water, 0.73 for NaCl and 0.54 mg/m^2^ for CaCl_2_) at pH 3 with or without ionic salts (Figure 4). The lower values clearly indicate the less availability of protein due to aggregation in an acidic medium. However, a remarkable increase in Г values was observed for pH 7 and 9. The most saturated layer was observed for pH 9 (4.11 for distilled water, 3.10 for NaCl and 4.61 mg/m^2^ for CaCl_2_). The emulsion droplet size and photograph (Figure 3A,E,F) are in correlation with Г (Figure 4) in this study, where a strong/dense matrix was observed for pH 7 and 9, probably because of a strong attraction between the aggregated and adsorbed protein of the interfacial layer. As discussed earlier, the solubility of protein is crucial for better emulsifying properties; therefore, the higher solubility of API at pH 7 and 9 with distilled water as well as with ionic salts compared to pH 3 shows higher Г values. Similar results for Г in the presence of ionic salts have been reported for myofibrillar protein stabilize emulsion [28]. The results further suggest that the presence of ionic salts does not resist the migration of protein to the interfacial layer.

### 3.5. Interfacial Layer Composition at SDS-PAGE

**Figure 5:**
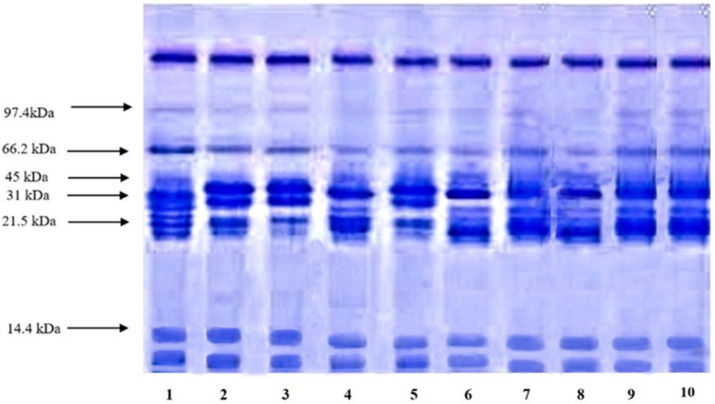
SDS-PAGE of absorbed API of 1% *w*/*v* emulsion. Lane 1 marker, lane 2 to lane 4 correspond to the interfacial composition of API in distilled water at pH 3, 7 and 9. Lane 5 to 7 present the interfacial composition of API in the presence of NaCl at pH 3, 7 and 9. Lane 8 to lane 10, interfacial composition of API in the presence of CaCl_2_ at pH 3, 7 and 9.

The interfacial layer composition of emulsion stabilized by API is shown in Figure 5. The gel was prepared with separating gel (15%) and 5% stacking gel to obtain the exact pattern of protein separation. The creamy layer was collected from the emulsions and was mixed with a 5 % sample solubilizing solution. The API shows seven major bands from 97.4 to 14.4 kDa (lane 2 and 3) at pH 3 and 7 without the addition of ionic salts while presenting six bands from 66.2 to 14.4 kDa for pH 9 (lane 4). However, the bands for lanes 2, 4 and 8 are not similar to that of native samples. The results clearly indicate the effects of pH and the type of ionic salt. A similar electrophoretic pattern of API was observed for lanes 2 and 5, along with a similar pattern for lanes 6 and 7. The most prominent fractions were observed around 45 to 21.5 kDa in all of the emulsions with and without ionic salts. These results indicate that ionic salts or change in pH might affect the migration of API to the interfacial layer. A similar electrophoretic pattern for API stabilized emulsion was reported by [7]. These results suggest that API was purely inserted in the interfacial layer and could stabilize the emulsion. Additionally, a few bands were demolished, and some new bands appeared during homogenization.

### 3.6. Emulsion Rheological Properties

#### 3.6.1. Rheological Behavior

The flow curve for emulsion was obtained between shear rate and shear stress, where the flow was calculated using the Herschel–Bulkley model (τ = τ_c_ + k·γ^n^). All of the emulsions present shear thinning behavior where *n* < 1 with and without ionic salts at different pH values. Further, all of the emulsions had almost a similar regression coefficient of sample fitting plot (R^2^) values, which indicates similar demolishing of emulsion under the applied stress. Additionally, the addition of salts at different pH values decreased the yield value (τ_c_). The decrease in yield value suggests emulsion decline towards Newtonian behavior under stress; however, no Newtonian characteristics were observed in all of the studied samples. The decrease in droplet sizes may affect the rheological behavior of emulsion; however, in this study, the higher volume frequency of larger droplet sizes (Figure 3B–D) is in conflict with the previous studies [29]. These results suggest that droplet sizes are not the only factor that could define the emulsion rheology. There are other factors, i.e., nature of the protein, medium of oil–water emulsions, volume frequency distribution and aggregation of the protein, that may also contribute to emulsion rheology.

#### 3.6.2. Flow Behavior (*n*)

The flow curves between shear rate and shear stress were obtained using Herschel–Bulkey’s model and various parameters are shown in Table 1. All of the emulsions with or without ionic salts present shear thinning behavior where τ_c_ > 0 and *n* < 1. The increase in flow curve was observed for all of the emulsions at pH 7 and 9, respectively. The *n* values of all the emulsions fall in the range of 0.51 to 0.91, which indicates their shear thinning behavior with or without salts. At pH 3 (distilled water), the *n* value is lower as compared to salt-treated samples. The same trend was repeated at pH 7 and 9, where a significant increase in the *n* value was noted with the addition of salts. This increase is a function of different salts added at different pHs.

Droplet size is a factor that could directly influence the flow behavior of emulsions [29]. However, in this study, larger droplet sizes (Figure 3A) were observed at pH 7 and 9 with or without ionic salts. These results indicate that there would be other factors, i.e., nature of the protein, viscosity of aqueous phase and interfacial protein composition, which would also control the flow behavior of emulsion [30].

#### 3.6.3. Viscosity

Viscosity was measured at a 125 s^−1^ shear rate for all of the emulsions. The emulsion of API in the presence of CaCl_2_ at pH 3 was observed to be more viscous (6.03 Pa s) compared to NaCl and distilled water. At pH 7, significantly higher viscosity (7.6 Pa s) was observed for distilled water followed by ionic salts, while at pH 9, higher viscosity (7.08 Pa s) was observed for CaCl_2_ (Table 1). Flocculation among oil droplets plays an important role in viscosity, i.e., higher flocculation leads to higher viscosity [31]. This hypothesis was true in the present study, where the emulsion photograph shows flocculation among oil droplets, particularly for pH 7 in the presence of NaCl (Figure 3C). However, other emulsions where there was no flocculation and higher viscosity were probably because of the strong network of the aggregated protein. Furthermore, the addition of ionic salts and different pH also influences the viscous behavior of emulsion.

## 4. Conclusions

The emulsifying and rheological properties of API were studied in acidic, neutral and alkali pH medium with or without adding ionic salts. The results clearly indicate the modification of protein structure where some of the emulsifying and rheological properties were observed depends on protein solubility in different pH mediums and the presence of ionic salts. A significant decrease was observed for EAI and ESI values with the addition of salts regardless of any pH medium or type of salt. Larger droplet sizes were observed for the emulsion prepared with API for salt added samples, with slight flocculation and dense matrix structure of emulsion. Significantly higher interfacial protein concentrations were observed at pH 9 with CaCl_2_. The SDS-PAGE analysis results demonstrate the actual migration of API to the interfacial layer and evident the presence of the full insertion of protein at the oil–water interface. All of the emulsion presents shear thinning behavior in a rheological study where the highest viscosity was observed for CaCl_2_ followed by NaCl and distilled water at pH 9, 7 and 3, respectively. In conclusion, the API was found to produce various emulsions with specific emulsifying and rheological behavior at different pH and ionic salts that could be translated to the industrial utilization of such proteins in food product development.

## Figures and Tables

**Table 1:** Modeling of the flow curves between 10 and 600 s^−1^ of shear stress of API emulsions at pH 3, 7 and 9 with and without NaCl and CaCl_2_.

	Herschel-Bulkey Factor	Distilled Water	Nacl	Cacl_2_
**pH 3**	*k* (Pa s^n^)	3.16 ± 0.73	7.09 ± 0.05	11.03 ± 1.62
	τ_c_ (Pa)	65.71 ± 0.001	91.67 ± 0.19	87 ± 0.22
	*n*	0.51 ± 0.023	0.77 ± 0.014	0.83 ± 0.65
	R^2^	0.9993 ± 0.0001	0.9991 ± 0.0001	0.9997 ± 0.0004
	η (Pa s) 125 s^−1^	5.9 ± 0.93	4.6 ± 0.44	6.03 ± 0.001
**pH 7**	*k* (Pa s^n^)	9.09 ± 0.001	4.19 ± 0.001	8.08 ± 0.001
	τ_c_ (Pa)	92.18 ± 0.21	63.59 ± 0.002	73.00 ± 0.001
	*n*	0.79 ± 0. 011	0.88 ± 0.001	0.91 ± 0.0.02
	R^2^	0.9994 ± 0.0001	0.9998 ± 0.0002	0.9994 ± 0.0001
	η (Pa s) 125 s^−1^	7.6 ± 0.44	4.8 ± 0.27	5.77 ± 0.001
**pH 9**	*k* (Pa s^n^)	6.07 ± 0.005	5.01 ± 0.001	7.09 ± 0.006
	τ_c_ (Pa)	94.93 ± 0.015	69.92 ± 0.001	77 ± 0.003
	*n*	0.87 ± 0. 001	0.93 ± 0.002	0.91 ± 0.001
	R^2^	0.9997 ± 0.0001	0.9997 ± 0.0001	0.9995 ± 0.0002
	η (Pa s) 125 s^−1^	6.1 ± 0.217	5.00 ± 0.002	7.08 ± 0.001

*k*: viscosity coefficient (Pa sn), τ_c_: yield value (mPa), n: flow index, R^2^: regression coefficient of sample fitting plot, η (mPa s): viscosity at a shear rate of 125 s^−1^.

## Data Availability

Not applicable.

## Sample Availability

Samples of the protein could be obtained by prior request to the author Nasir Mehmood Khan.

## References

[1] Zhang W., Liu C., Zhao J., Ma T., He Z., Huang M., Wang Y. (2021). Modification of structure and functionalities of ginkgo seed proteins by pH-shifting treatment. Food Chem..

[2] De Silva A.M.M., Almeida F.S., Sato A.C.K. (2021). Functional characterization of commercial plant proteins and their application on stabilization of emulsions. J. Food Eng..

[3] Zhou L., Zhang J., Xing L., Zhang W. (2021). Applications and effects of ultrasound assisted emulsification in the production of food emulsions: A review. Trends Food Sci. Technol..

[4] Tang Y.R., Ghosh S. (2021). Stability and rheology of canola protein isolate-stabilized concentrated oil-in-water emulsions. Food Hydrocoll..

[5] Morales D. (2021). Oak trees (*Quercus* spp.) as a source of extracts with biological activities: A narrative review. Trends Food Sci. Technol..

[6] Taib M., Bouyazza L. (2021). Composition, physicochemical properties, and uses of Acorn starch. J. Chem..

[7] Aziz A., Nasir M.K., Farman A., Zia U.K., Shujaat A., Abdul K.J., Noor R., Nawshad M. (2020). Effect of protein and oil volume concentrations on emulsifying properties of acorn protein isolate. Food Chem..

[8] Tejerina D., García-Torres S., de Vaca M.C., Vázquez F.M., Cava R. (2011). Acorns (*Quercus rotundifolia* Lam.) and grass as natural sources of antioxidants and fatty acids in the “montanera” feeding of Iberian pig: Intra-and inter-annual variations. Food Chem..

[9] Szyndler-Nędza M., Świątkiewicz M., Migdał Ł., Migdał W. (2021). The Quality and Health-Promoting Value of Meat from Pigs of the Native Breed as the Effect of Extensive Feeding with Acorns. Animals.

[10] Saxena J., Adhikari B., Brkljaca R., Huppertz T., Zisu B., Chandrapala J. (2021). Influence of Lactose on the Physicochemical Properties and Stability of Infant Formula Powders: A Review. Food Rev. Int..

[11] Williams S., AOAC (1984). Methods 932.06, 925.09, 985.29, 923.03. Official Methods of Analysis of the AOAC.

[12] Casella M.L.A., Whitaker J.R. (1990). Stabilization of proteins by solvents. J. Food Biochem..

[13] Peterson G.L. (1977). A simplification of the protein assay method of Lowry et al., which is more generally applicable. Anal. Biochem..

[14] Markwell M.A.K., Haas S.M., Bieber L.L., Tolbert N.E. (1978). A modification of the lowry procedure to simplify protein determination in membrane and lipoprotein samples. Anal. Biochem..

[15] Pearce K.N., Kinsella J.E. (1978). Emulsifying properties of protein: Evaluation of a turbidimetric technique. J. Agric. Food Chem..

[16] Walstra P., Becher P. (1983). Formation of Emulsion. Encyclopedia of Emulsion Technology: Basic Theory.

[17] Patton S., Huston G.E. (1986). A method for isolation of milk fat globules. Lipids.

[18] Laemmli U.K. (1970). Cleavage of structural proteins during the assembly of the head of bacteriophage T4. Nature.

[19] Vojdani J. (1996). Solubility. Methods of Testing Protein Functionality.

[20] Nahar M., Zakaria Z., Hashim U., Bari M. (2017). Effect of pH and salt concentration on protein solubility of slaughtered and non-slaughtered broiler chicken meat. Sains Malays..

[21] Mu T.H., Tan S.S., Xue Y.L. (2009). The amino acid composition, solubility and emulsifying properties of sweet potato protein. Food Chem..

[22] El Nasri N.A., El Tinay A.H. (2007). Functional properties of fenugreek (*Trigonella foenum* graecum) protein concentrate. Food Chem..

[23] Sarkar A., Singh H. (2016). Emulsions and foams stabilized by milk proteins. Advanced Dairy Chemistry.

[24] Dickinson E. (1998). Proteins at interfaces and in emulsions stability, rheology and interactions. J. Chem. Soc. Farad. Trans..

[25] Ebert S., Grossmann L., Hinrichs J., Weiss J. (2019). Emulsifying properties of water-soluble proteins extracted from the microalgae *Chlorella sorokiniana* and *Phaeodactylum tricornutum*. Food Funct..

[26] Zhong Y., Zhao J., Dai T., Ye J., Wu J., Chen T., Liu C. (2021). Fabrication of Oil-in-Water emulsions with whey protein isolate–puerarin composites: Environmental stability and interfacial behavior. Foods.

[27] Li M., McClements D.J., Liu X., Liu F. (2020). Design principles of oil-in-water emulsions with functionalized interfaces: Mixed, multilayer, and covalent complex structures. Compr. Rev. Food Sci. Food Saf..

[28] Guo X., Zhang Y., Jamali M.A., Peng Z. (2021). Manipulating interfacial behavior and emulsifying properties of myofibrillar proteins by L-Arginine at low and high salt concentration. Int. J. Food Sci. Technol..

[29] Pal R. (1996). Effects of droplet size on the rheology of emulsions. AIChE J..

[30] Drusch S., Klost M., Kieserling H. (2021). Current knowledge on the interfacial behaviour limits our understanding of plant protein functionality in emulsions. Curr. Opin. Colloid. Interface Sci..

[31] Khan N.M., Mu T.H., Zhang M., Arogundade L.A. (2014). The effects of pH and high hydrostatic pressure on the physicochemical properties of a sweet potato protein emulsion. Food Hydrocoll..

